# Left Atrium and Left Atrial Appendage Remodeling Assessed by Echocardiography Predict Atrial Fibrillation Recurrence After Radiofrequency Catheter Ablation

**DOI:** 10.1002/clc.70457

**Published:** 2026-09-02

**Authors:** Binyang Zhu, Shaohui Qu, Hairu Li, Dandan Yu, Yingying Liu, Xin Ai, Guangyin Li, Shuangquan Jiang

**Affiliations:** ^1^ Department of Ultrasound The Second Affiliated Hospital of Harbin Medical University Harbin City Heilongjiang Province China

**Keywords:** atrial fibrillation, left atrial appendage strain, left atrial strain, radiofrequency catheter ablation, recurrence

## Abstract

**Purpose:**

The aim of this study is to evaluate left atrium (LA) and left atrial appendage (LAA) remodeling using two‐dimensional speckle tracking echocardiography (2D‐STE) and investigate its predictive value for atrial fibrillation (AF) recurrence after radiofrequency catheter ablation (RFCA).

**Methods:**

One hundred patients with AF underwent TTE and TEE before undergoing RFCA. LA and LAA strain and strain rate values obtained by 2D‐STE. Logistic regression analysis was used to analyze independent contributors for AF recurrence after RFCA. The prediction efficiency of clinical ultrasound parameter models associated with the presence of AF recurrence was evaluated by the receiver operating characteristic (ROC) curve with area under curve (AUC).

**Results:**

①During the 1‐year follow‐up, 33% of patients experienced AF recurrence. ②The recurrent group had significantly higher NLR, NT‐proBNP, and LAVImin values compared to the non‐recurrent group (*p* < 0.05). ③The recurrence group had significantly lower LAEF, LAEI, LAA‐EV, LAS, LASR, LAAS, LAASR values than the non‐recurrent group (*p* < 0.05). ④LAAS and LAASR were potential factors contributing to AF recurrence (*p* < 0.05). In addition, incorporating LAAS and LAASR into predictive models can provide extra valuable information to identify patients at a higher risk of AF recurrence.

**Conclusion:**

LAAS and LAASR, as obtained by 2D‐STE, are potential predictors of AF recurrence after RFCA. By incorporating these 2D‐STE parameters into preoperative clinical data along with TTE and TEE, it may be possible to identify impaired LA/LAA function in AF patients early. This could aid in choosing suitable patients and improving the benefit ratio of RFCA.

## Graphical abstract

LAAS and LAASR were independent factors contributing to AF recurrence. Incremental value of LAAS and LAASR in risk prediction of AF recurrence.

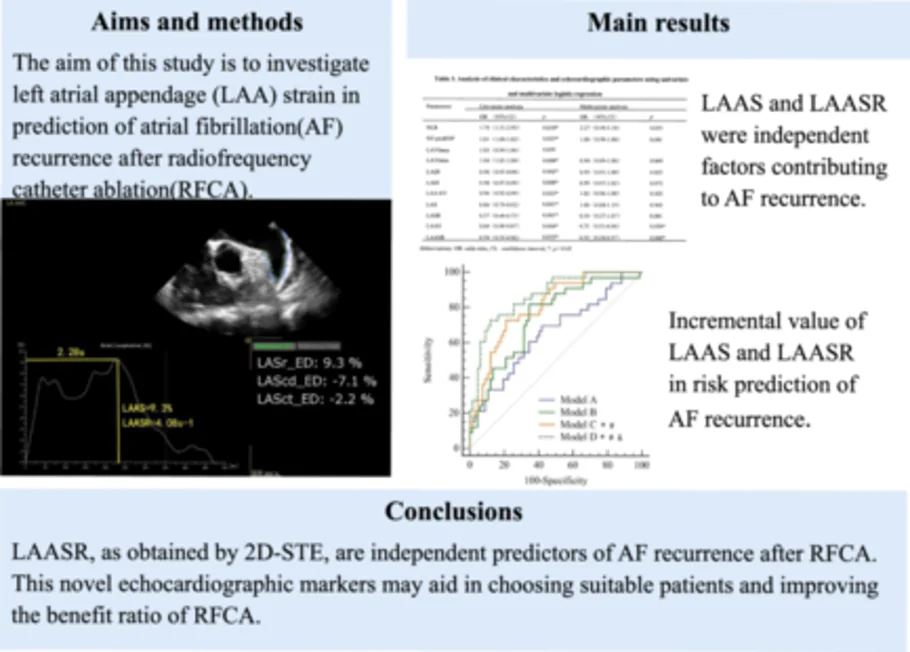

## 1 Introduction

Atrial fibrillation (AF) is a common cardiac arrhythmia, with a prevalence of 2%–4% [1] in the general population. The prevalence of AF is rising worldwide and is expected to triple by 2030 [2, 3], due to the aging population and better urvival rates for patients with chronic cardiovascular diseases. AF is associated with a poor prognosis in heart failure and myocardial infarction. Additionally, it is a significant risk factor for thrombosis and embolic stroke. More than 60% of patients with AF endure a significant reduction in quality of life and exercise tolerance. They also face an elevated risk of hospitalization, which imposes a substantial burden on public health [4].

Strategies for managing patients with AF include controlling the ventricular rate, restoring and maintaining sinus rhythm. As a first‐line therapy, radiofrequency catheter ablation (RFCA) [5, 6] is more effective than antiarrhythmic drugs at maintaining sinus rhythm for AF patients. It can also significantly improve patients' symptoms and quality of life. Circumferential pulmonary vein isolation forms the basis of RFCA, yet recurrence rates still remain high, ranging from 25% to 40% [6, 7, 8].

AF is a progressive disease with complex pathophysiological mechanisms related to remodeling of the left atrium (LA) and left atrial appendage (LAA) [9, 10]. These mechanisms involve atrial dilatation, myocardial fibrosis, and myocardial dysfunction. Particularly, myocardial fibrosis plays a crucial role in the maintenance and persistence of AF. Left atrial strain (LAS), obtained by two‐dimensional speckle tracking echocardiography (2D‐STE), is a non‐invasive indicator of myocardial deformation. This simple and reproducible tool assesses LA function, reflecting both structural remodeling and dysfunction of the LA. Numerous studies [11, 12, 13] have discovered a significant correlation between LAS and the degree of LA fibrosis, which is a crucial factor in the AF recurrence after RFCA [14]. Previous study [15] has shown that LAA structural and functional parameters (e.g. volume, orifice area, flow velocity) are linked with AF recurrence. However, evidence regarding LAAS, an emerging parameter that directly quantifies the deformation capacity of LAA myocardial fibers, remains limited. The aim of this study was to determine the predictive value of LA and LAA remodeling assessed by 2D‐STE in the AF recurrence after RFCA. Such evaluation can assist in identifying patients who may benefit from RFCA treatment.

## 2 Materials and Methods

### 2.1 Study Population

172 patients with AF who underwent RFCA for the first time in the Department of Cardiology of the Second Affiliated Hospital of Harbin Medical University between April 2022 and December 2022 were admitted to this research. We excluded 72 patients either with congenital heart disease (*n* = 2), organic valvular heart disease (*n* = 6), previous pacemaker implantation (*n* = 10), LA/LAA spontaneous echo contrast or thrombosis as detected by transesophageal echocardiography (TEE) (*n* = 15), loss of follow‐up (*n* = 20) and ultrasound images of poor quality that cannot be quantitatively analyzed (*n* = 19). In addition, we excluded patients with low ejection fraction (< 50%) from beginning. Finally, a total of 100 patients with AF were included in this study. A 12‐lead electrocardiogram or 24 h‐Holter recording was utilized for the diagnosis of AF. All patients received RFCA treatment and were monitored as outpatients for 12 months. The study was approved by the ethics committee of the hospital, and all participants signed an informed consent form.

### 2.2 Baseline Clinical Characteristics

Clinical characteristics included age, gender, body mass index (BMI), medical history, preoperative laboratories results, medications, as well as detailed records of the AF characteristics. The later consisted of the type of AF, CHA_2_DS_2_‐VASc score, and HAS‐BLED score. Additionally, the CHA_2_DS_2_‐VASc risk score is used to predict the risk of thromboembolism in AF patients. The HAS‐BLED scoring system, on the other hand, was designed to evaluate the risk of major bleeding in patients taking anticoagulants. Those with a score of 3 or higher are considered high‐risk for major bleeding and should be cautious when using anticoagulants.

### 2.3 Echocardiography

#### 2.3.1 Transthoracic Echocardiography and Transesophageal Echocardiography

The Philips EPIQ 7C heart ultrasound system (Netherlands) was used to complete the preoperative transthoracic echocardiography (TTE) and TEE examinations of all patients within 3 days prior to RFCA procedure by experienced sonographers. The patient was lying on the left side in a resting state, connected to a 12‐lead ECG for transthoracic image acquisition. The probe was a cardiac probe (X5‐1, X7‐2t) and an average frame rate of 50 frames/s. Standard M‐shaped and 2D images were obtained by operating near the sternum and apex. The left ventricular ejection fraction (LVEF) was evaluated using the biplane Simpson method by echocardiography. Volumetric parameters of the LA were evaluated using the Simpson method, which calculates the LA volume divided by body surface area. All patients completed TEE and pulse Doppler measurement of the LAA emptying velocity (LAA‐EV) and filling velocity (LAA‐FV). LAA was scanned in multiple mid‐esophageal imaging planes from 0° to 180° to optimize the visualization of the entire LAA. These measurements were taken at the central position, less than 1 cm from the entrance of LAA. Two experienced doctors performed the image acquisition, and five cardiac cycles were continuously acquired. This approach was applied consistently across all patients irrespective of rhythm status at the time of echocardiographic examination.

#### 2.3.2 2D Speckle Tracking Echocardiography

The Philips QLAB 13.0 software was used to analyze the dynamic images of LA and LAA. Strain analysis was performed with reference to the 2018 consensus for the standardization of myocardial strain imaging [16], using left ventricular end‐diastole as the zero baseline.

LA strain‐time curves were generated by first automatically identifying the LA endocardium and manually adjusting the region of interest (ROI) range to avoid the LAA and pulmonary vein openings. This is achieved by using the AutoStrain LA function of the program on both the apical two‐chamber and apical four‐chamber view. This ensures the tracing line precisely fits the endocardial line throughout the cardiac cycle. Left atrial strain (LAS) is calculated as the difference between the strain value at end‐diastole of the left ventricle and the strain value at the mitral valve opening. The peak time is measured as the duration from the zero baseline to the peak of the strain curve. Left atrial strain rate (LASR) is then calculated as LAS divided by the peak time. The echocardiographic images of five cardiac cycles were analyzed and averaged to obtain the final LAS and LASR values.

In QLAB AutoStrain LA mode, we manually tracked LAA endocardium by using three reference points at the LAA views, the first point placed at the medial bottom, the second point at the lateral bottom, and the third point at the LAA roof. Then, the region of interest was automatically created. On this basis, we manually adjusted the region of interest to cover the entire wall of LAA. During the analysis, the operator observed whether the region of interest moved along the endocardial boundary of LAA during the whole cardiac cycle and made appropriate adjustments. LAAS is calculated as the difference between the strain value at end‐diastole of the left ventricle and the strain value at the mitral valve opening. The peak time is measured as the duration from the zero baseline to the peak of the strain curve. LAASR is then calculated as LAAS divided by the peak time. The echocardiographic images of five cardiac cycles were analyzed and averaged to obtain the final LAAS and LAASR values. The analysis of echocardiographic data was performed by the same operator with strain imaging experience and without knowledge of patient information.

### 2.4 RFCA Procedures

The RFCA procedure was performed under local anesthesia. The coronary sinus electrode was delivered via a puncture to the left femoral vein. The right femoral vein was punctured twice, followed by the insertion of the 11F sheath and the SL‐1 sheath. All patients underwent pulmonary vein angiography, and a real‐time three‐dimensional electro‐anatomic LA map was created by the CARTO 3 mapping system. Bilateral linear ablation of the pulmonary veins was performed. Following this, a Pentaray catheter was introduced to the LA for agonist and stromal marking. Any detected leakage points were repaired and ablated as required. The endpoints for the RFCA were AF terminated, and sinus rhythm restored. A 30 min of postoperative observation is mandatory, with the procedure deemed successful if there is no recovery of pulmonary vein potentials.

### 2.5 Patient Follow‐Up and AF Recurrence

After hospital discharge, all patients were scheduled in our outpatient clinic 3, 6 and 12 months after ablation. Furthermore, patients were asked for procedure complications, any arrhythmia‐related symptoms, any documented arrhythmia recurrences, and current medication. For patients with chest tightness, palpitations, or conscious recurrence of AF, they are advised to immediately return to the hospital and undergo ECG to determine whether there was AF recurrence. AF recurrence after RFCA was defined as an atrial arrhythmia (atrial tachycardia, atrial flutter, AF) with a duration of at least 30 s documented on an ECG after the 3‐month postoperative blanking period for RFCA. If the above ECG changes were not recorded during the 1‐year follow‐up, the patient was considered to have no AF recurrence.

### 2.6 Statistical Analysis

Data analysis was conducted using SPSS 26.0 and MedCalc 20.022 statistical software. Continuous variables are expressed as mean ± SD, and categorical variables were expressed as count and percentage. Data were compared using the Fisher *t* test or chi‐square test for categorical variables and the Student *t* test or Mann–Whitney *U* test for continuous variables, where appropriate. Univariate and multivariate logistic regression analysis were used to identify the independent contributors affecting AF recurrence after RFCA, and the prediction models based on clinical and echocardiographic data were established to analyze the incremental value of each independent contributor. The prediction efficiency of factors associated with AF recurrence was evaluated by receiver operating characteristic (ROC) curve with area under curve (AUC). All statistical tests were two‐sided, and a *P* value less than 0.05 was considered statistically significant.

## 3 Results

### 3.1 Study Cohort

A total of 100 patients (59.6 ± 9.8 years, 66% males, 34% females) were enrolled in this cohort. During the 1‐year follow‐up, 33 patients experienced a recurrence and 67 did not. Table 1 shows the demographic characteristics, medical history, preoperative laboratory data, and medications of the 2 groups. Compared to the non‐recurrent group, the recurrent group had a higher level of NT‐proBNP (*p* = 0.015) and a higher NLR (*p* = 0.028). Further, there were no differences in the age, gender, medications, and comorbidities, gender, medications, and comorbidities, between these groups.

**Table 1:** Clinical characteristics of the study population.

Parameters	Recurrence group (*n* = 33)	Non‐recurrence group (*n* = 67)	t/χ2/Z	*p*
Demographics				
Age (year)	59.0 (53.5–65.0)	60.0 (55.0–66.0)	−0.514	0.607
Male, *n* (%)	20 (60.6%)	46 (68.7%)	0.639	0.424
BMI (kg/m2)	26.1 ± 3.4	24.9 ± 2.9	−1.839	0.069
AF characteristics				
Persistent AF, *n* (%)	18 (54.5%)	30 (44.8%)	0.845	0.358
AF duration (month)	24.1 (11.5–42.0)	12.2 (7.2–48.0)	−1.322	0.186
CHA2DS2VASc score	1 (0–3)	1 (1–2)	−0.513	0.608
HASBLED score ≥ 3	0 (0%)	6 (9.0%)	/	0.174#
Medical history, *n* (%)				
Stroke	5 (15.2%)	12 (17.9%)	0.119	0.730
Coronary artery disease	7 (21.2%)	8 (11.9%)	1.491	0.222
Hypertension	12 (36.4%)	28 (41.8%)	0.271	0.602
Diabetes mellitus	6 (18.2%)	9 (13.4%)	0.391	0.532
Hyperlipidemia	13 (39.4%)	32 (47.8%)	0.625	0.429
Laboratories				
NT‐proBNP, pg/mL	382.0 (76.5–710.1)	117.1 (48.2–462.1)	−2.43	0.015*
Creatinine, μmol/L	81.6 (68.5–89.5)	81.3 (72.1–88.4)	−0.051	0.959
NLR	2.6 ± 1.0	2.2 ± 0.7	−2.223	0.028*
Medications, *n* (%)				
Amiodarone	25 (75.8%)	39 (58.2%)	2.955	0.086
Propafenone	1 (3.0%)	4 (6.0%)	0.402	0.526
Anticoagulation drugs	31 (93.9%)	58 (86.6%)	1.227	0.268
Beta blocker	12 (36.4%)	17 (25.4%)	1.297	0.255
Calcium channel blockers	4 (12.1%)	16 (23.9%)	1.911	0.167
Statins	24 (72.7%)	45 (67.2%)	0.320	0.572
ACEI/ARB	21 (63.6%)	37 (55.2%)	0.642	0.423

Abbreviations: ACEI/ARB, Angiotensin‐converting enzyme inhibitor/angiotensin receptor blocker; AF, atrial fibrillation; BMI, body mass index; NLR, neutrophil to lymphocyte ratio; NT‐proBNP, *N*‐terminal pro‐B‐type natriuretic peptide.

* *p *＜ 0.05.

# Fisher's exact test.

### 3.2 Echocardiographic Parameters

**Figure 1:**
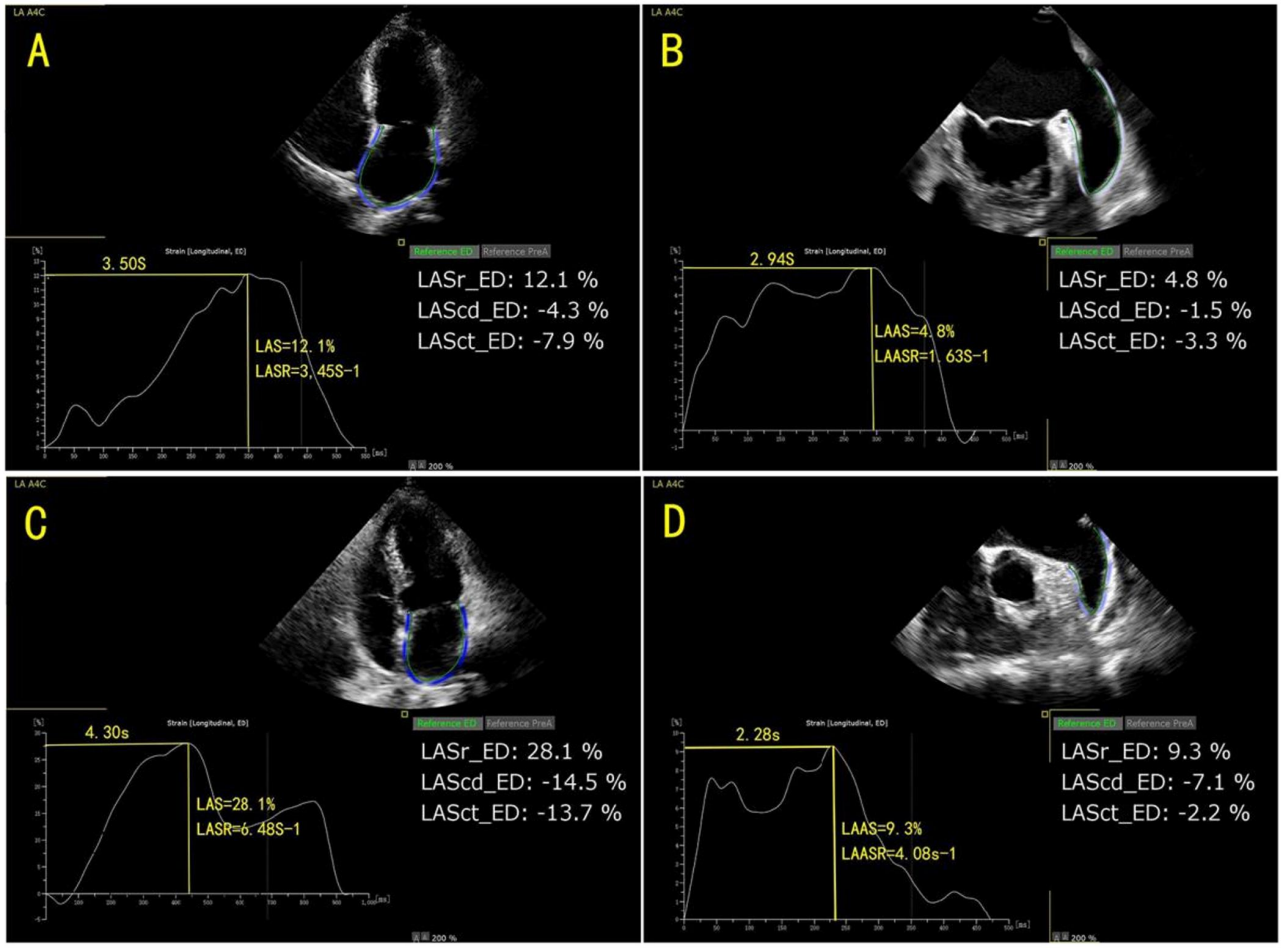
Schematic diagram of strain analysis between patients in the recurrence group and the non‐recurrence group. A and B show the same patients in the recurrence group, LAS = 12.1%, LASR = 3.45s‐1; LAAS = 4.8%, LAASR = 1.63s‐1; C and D show the same patients in the non‐recurrence group, LAS = 28.1%, LASR = 6.48s‐1; LAAS = 9.3%, LAASR = 4.08s‐1.

The echocardiographic parameters of the two groups are shown in Table 2. Upon comparison, the recurrent group exhibited higher LAVImax and LAVImin than the non‐recurrent group. Conversely, LAEF, LAEI, and LAA‐EV were lower in the recurrent group. All these differences were being statistically significant (*p* < 0.05). The differences in LVEF, E, E/e', LAD, and LAA‐FV between the two groups were not statistically significant (*p* ＞ 0.05). When comparing 2D‐STE parameters, LAS, LASR, LAAS, and LAASR were lower in the recurrent group than in the non‐recurrent group, and the differences were all statistically significant (*p* < 0.001), as shown in Table 2. The LA and LAA strain analysis for both groups is shown in Figure 1.

**Table 2:** Comparison of echocardiographic parameters between patients in the recurrence group and the non‐recurrence group.

Parameters	Recurrence group (*n* = 33)	Non‐recurrence group (*n* = 67)	t/χ2/Z	*p*
LVEF (%)	64.9 (60.9–66.6)	64.5 (62.5–66.4)	−0.510	0.610
E (cm/s)	78.1 (57.2–95.0)	69.8 (61.1–87.8)	−1.037	0.300
E/e'	8.9 (6.7–11.9)	8.9 (6.7–11.2)	−0.480	0.631
LAD (mm)	41 (38.6–45.0)	39.7 (36.6–43.8)	−1.492	0.136
LAVImax (mL/m^2^)	38.6 (33.9–49.4)	32.4 (26.1–42.6)	−2.621	0.009*
LAVImin (mL/m^2^)	29.6 (22.7–37.4)	19.2 (14.9–25.6)	−3.772	0.001*
LAEF (%)	26.9 ± 15.4	38.5 ± 16.8	−3.337	0.001*
LAEI (%)	32.0 (20.5–64.5)	69.1 (31.0–110.2)	−3.219	0.001*
LAS (%)	12.9 (8.3–19.4)	22.4 (15.3–26.1)	−4.593	0.001*
LASR (s^−1^)	3.8 (2.5–4.8)	6.1 (4.5–7.1)	−4.809	0.001*
LAA‐EV (cm/s)	42.6 (33.1–52.8)	52 (39.9–56.0)	−2.254	0.024*
LAA‐FV (cm/s)	44.0 ± 11.7	46.0 ± 9.9	0.910	0.365
LAAS (%)	4.3 (3.3–5.5)	6.6 (4.9–8.9)	−4.824	0.001*
LAASR (s^−1^)	2.3 (1.2–2.8)	3.2 (2.3–4.1)	−4.003	0.001*

Abbreviations: E, the peak early‐diastolic flow velocity of mitral valve; e', the average of lateral and septal mitral annular early‐diastolic peak velocity, LAA‐EV, left atrial appendage emptying velocity; LAA‐FV, left atrial appendage filling velocity; LAAS, left atrial appendage strain; LAASR, left atrial appendage strain rate; LAD, left atrial diameter; LAEF, left atrial emptying fraction; LAEI, left atrial expansion index; LAS, left atrial strain; LASR, left atrial strain rate; LAVImax, maximum left atrial volume index, LAVImin, minimum left atrial volume index; LVEF, left ventricular ejection fraction.

* *p* ＜ 0.05.

### 3.3 Factors Associated With Recurrence After RFCA in Patients With AF and Their Diagnostic Value

Clinical characteristics and echocardiographic parameters associated with AF recurrence were assessed using univariate logistic regression analysis. The results showed that NLR, NT‐proBNP, LAVImin, LAEF, LAEI, LAA‐EV, LAS, LASR, LAAS, and LAASR were significant factors for AF recurrence after RFCA. Higher NLR, NT‐proBNP, and LAVImin were identified as risk factors for AF recurrence. Meanwhile, higher LAEF, LAEI, LAA‐EV, LAS, LASR, LAAS and LAASR were protective factors against AF recurrence. After conducting a multivariable regression analysis of these 10 factors, only LAAS (OR: 0.71, *p* < 0.05) and LAASR (OR: 0.53, *p* < 0.05) were identified as potential predictors of AF recurrence after RFCA. These findings are illustrated in Table 3.

**Table 3:** Analysis of clinical characteristics and echocardiographic parameters using univariate and multivariate logistic regression.

Parameters	Univariate analysis		Multivariate analysis	
	OR (95% CI)	*p*	OR (95% CI)	*p*
NLR	1.74 (1.31–2.95)	0.038*	2.27 (0.99–5.18)	0.053
NT‐proBNP	1.01 (1.00–1.02)	0.023*	1.00 (0.99–1.00)	0.681
LAVImax	1.03 (0.99–1.06)	0.059		
LAVImin	1.04 (1.01–1.08)	0.008*	0.94 (0.89–1.00)	0.069
LAEF	0.96 (0.93–0.98)	0.002*	0.99 (0.91–1.08)	0.823
LAEI	0.98 (0.97–0.99)	0.008*	0.99 (0.97–1.02)	0.872
LAA‐EV	0.96 (0.92–0.99)	0.022*	1.02 (0.96–1.08)	0.425
LAS	0.86 (0.79–0.92)	0.001*	1.00 (0.84–1.19)	0.965
LASR	0.57 (0.44–0.75)	0.001*	0.54 (0.27–1.07)	0.081
LAAS	0.66 (0.49–0.87)	0.004*	0.71 (0.51–0.98)	0.036*
LAASR	0.58 (0.35–0.96)	0.035*	0.52 (0.28–0.97)	0.040*

Abbreviations: CI, confidence interval; OR, odds ratio.

* *p *＜ 0.05.

To compare the diagnostic value of each factor in predicting the risk of AF recurrence after RFCA, ROC curves were plotted for 10 parameters: NLR, NT‐proBNP, LAVImin, LAEF, LAEI, LAA‐EV, LAS, LASR, LAAS, and LAASR. Their diagnostic efficacy was recorded respectively. Among them, LAVImin > 22.8 mL/m^2^ (AUC = 0.73, *p* < 0.001), LAS ≤ 22% (AUC = 0.78, *p* < 0.001), LASR ≤ 5.32 s^−1^ (AUC = 0.76, *p* < 0.001), LAAS ≤ 5.67% (AUC = 0.79, *p* < 0.001), LAASR ≤ 2.70 s^−1^ (AUC = 0.75, *p* < 0.001) exhibited better diagnostic efficacy for AF recurrence, as shown in Table 4.

**Table 4:** Univariate analysis for the prediction of AF recurrence.

Parameters	AUC	95%CI	Cut‐off value	Sensitivity	Specificity	Youden index	*p*
NLR	0.61	0.51–0.71	> 1.79	84.8%	34.3%	0.19	0.058
NT‐proBNP	0.65	0.55–0.74	> 143 pg/mL	69.7%	58.2%	0.28	0.012*
LAVImin	0.73	0.63–0.82	> 22.8 mL/m^2^	75.7%	68.6%	0.44	< 0.001*
LAEF	0.69	0.59–0.78	≤ 30%	69.7%	67.1%	0.37	< 0.001*
LAEI	0.69	0.59–0.78	≤ 42%	69.7%	68.6%	0.38	< 0.001*
LAA‐EV	0.63	0.54–0.73	≤ 46.4 cm/s	66.6%	59.7%	0.26	0.022*
LAS	0.78	0.69–0.86	≤ 22%	90.9%	53.7%	0.44	< 0.001*
LASR	0.76	0.67–0.84	≤ 5.32 s^−1^	84.8%	64.1%	0.49	< 0.001*
LAAS	0.79	0.70–0.87	≤ 5.67%	84.8%	64.1%	0.49	< 0.001*
LAASR	0.75	0.65–0.83	≤ 2.70 s^−1^	75.7%	62.6%	0.38	< 0.001*

Abbreviations: AUC, the area under the curve; CI, confidence interval.

* *p *＜ 0.05.

### 3.4 Diagnostic Efficacy of a Combined Model Incorporating Clinical Characteristics and Echocardiographic Parameters

Four prediction models were developed based on various combinations of clinical characteristics and echocardiographic parameters: Model A (NLR, NT‐proBNP); Model B (incorporates Model A and adds LAVImin, LAEF, LAEI); Model C (builds on Model B and adds LAS, LASR); and Model D (extends Model C by adding LAA‐EV, LAAS, LAASR). These are detailed in Table 5.

**Table 5:** Comparing of the efficacy of the combined diagnostic model of clinical characteristics and echocardiographic parameters.

Model	AUC	95%CI	Sensitivity	Specificity	*p*
Model A	0.65	0.55–0.75	69.7%	58.2%	0.009*
Model B	0.74	0.64–0.83	81.8%	65.6%	< 0.001*
Model C	0.81	0.72–0.88	72.7%	79.1%	< 0.001*
Model D	0.86	0.78–0.92	72.7%	88.1%	< 0.001*

*Note:* Model A (NLR、NT‐proBNP), Model B (Model A+ LAVImin、LAEF、LAEI), Model C (Model B + LAS、LASR), Model D (Model C + LAA‐EV、LAAS、LAASR):

Abbreviations: AUC, the area under the curve; CI, confidence interval.

* *p *＜ 0.05.

**Figure 2:**
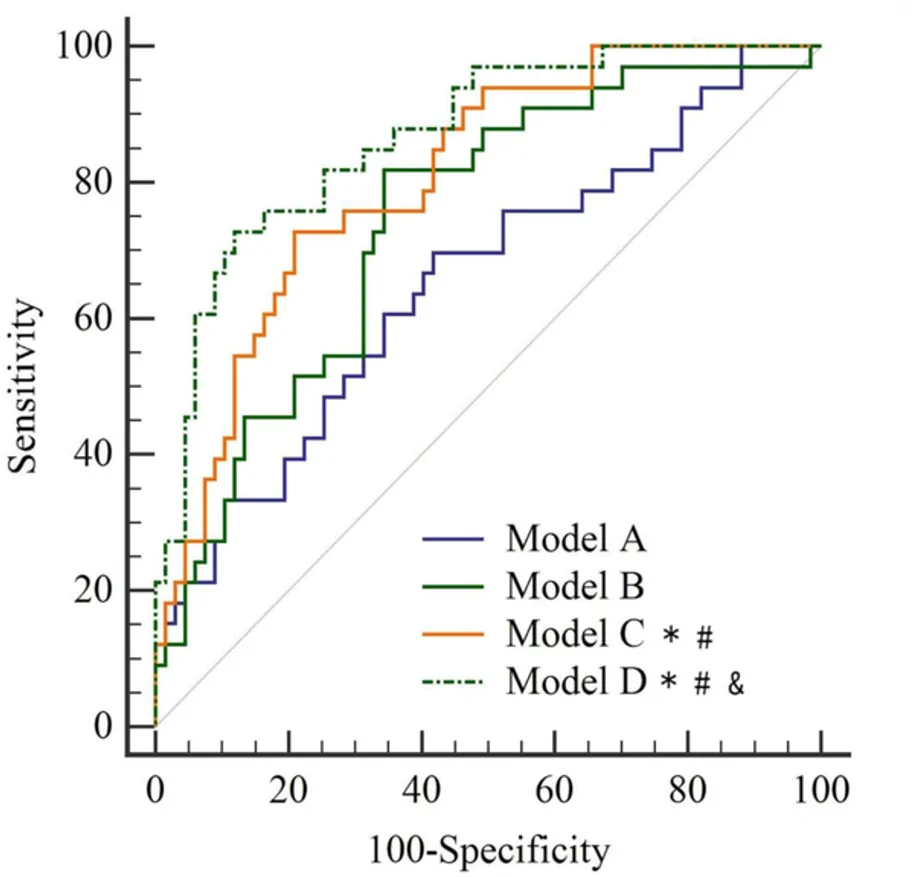
ROC curve of the combined diagnostic model predicting AF recurrence after RFCA. *p < 0.05 compared to Model A, ^#^ *p* < 0.05 compared to Model B, ^&^ *p* < 0.05 compared to Model C.

The diagnostic efficacy of the four models was evaluated and compared. Model D exhibited the best diagnostic efficacy (AUC = 0.86, *p* < 0.05). Model C also demonstrated significantly better diagnostic efficacy than Model A and Model B (*p* < 0.05), as illustrated in Figure 2.

## 4 Discussion

The present study analyzed 100 patients with AF who underwent RFCA for the first time. The general clinical and echocardiographic data of the patients were investigated before RCFA and the findings revealed that the LAAS and LAASR were potential predictors of AF recurrence. A model combining clinical characteristics and echocardiographic parameters (NT‐proBNP, NLR, LAVImin, LAEF, LAEI, LAS, LASR, LAA‐EV, LAAS, and LAASR) showed superior diagnostic performance (AUC = 0.86) and higher specificity (88.1%). The added value of our study lies not in re‐demonstrating the predictive role of LAA strain, but in developing and validating a multiparametric model (Model D) that integrates LAAS with complementary functional parameters—including LAAEV, LAS, and other conventional echocardiographic indices. Our results demonstrate that this integrated approach achieves superior predictive specificity compared to single‐parameter models, potentially reducing false‐positive rates and improving clinical decision‐making in risk stratification.

Relevant studies have demonstrated that the rate of recurrence of AF following RFCA is 25%–40% [6, 7, 8]. In this study, 33% of the patients had a recurrence, a rate consistent with those reported in previous studies [7, 8]. Despite extensive efforts to detect asymptomatic AF events, true recurrence rates may have been underestimated by a lack of continuous AF monitoring during the follow‐up period. AF is a progressive disease principally characterized by inflammation, atrial dilatation, and myocardial fibrosis. As the gold standard for assessing atrial fibrosis, late gadolinium enhancement magnetic resonance imaging (LGE‐MRI) has demonstrated that atrial fibrosis burden is a significant independent risk factor for long‐term AF recurrence after RFCA. The LAA is a remnant of the original embryonic LA that develops during the third week of gestation and is an important accessory structure to the LA [17]. Suksaranjit [18] found that severe LAA fibrosis increases the risk of AF recurrence by approximately four times. This is in line with the LAA's crucial electrophysiological function in sustaining AF. In a study of patients undergoing secondary ablation, Di Biase et al. [19] identified the LAA as the sole source of AF recurrence in 86 (8.7%) cases, with no pulmonary vein or extrapulmonary triggers detected. Higher AF recurrence after RFCA could be attributed to non‐pulmonary venous ectopic lesions, and the LA and LAA are ectopic pacemakers for AF other than pulmonary vein, structural remodeling in these regions progresses during AF, and is especially pronounced when ectopic activity originates within the LA or LAA [20]. Previous studies on AF recurrence determinants have emphasized the LAS over the LAAS.

Although increased LAVImax, LAVImin and reduced LAEF, LAEI differed significantly between recurrence and non‐recurrence group and showed predictive trends, they were not independent predictors. LA strain can detect early atrial dysfunction. Furthermore, LAS and LASR have been identified as important factors influencing recurrence after RFCA [21, 22]. The study by Liżewska‐Springer [23] concluded that LAS < 20%–25% was associated with AF recurrence, which was in agreement with the findings obtained herein(LAS < 22%). LA remodeling increases LA size, pressure, and volume. LAA dilation promotes greater atrial natriuretic peptide secretion than LA dilation [24, 25], leading to increased urine volume and urinary sodium. This mechanism helps regulate LA pressure by via reservoir function. However, an enlarged and dysfunctional LAA reduces and stagnants blood flow, reflected by lower LAA‐EV. Multiple studies [26, 27, 28] show that low LAA‐EV independently predicts AF recurrence after ablation. Reduced LAA‐EV may indicate early functional LA or LAA remodeling in AF, potentially preceding LA dilation. Our study found LAA‐EV ≤ 46.4 cm/s as a risk factor for AF recurrence after RFCA, though with limited diagnostic efficacy (AUC = 0.63), consistent with Istratoaie's finding (cutoff: 40.5 cm/s) [27].

Previous studies on AF recurrence have placed greater focus on LAA structure (LAA volume, orifice area, orifice long/short axis, and volume index), LAA function (LAA emptying flow velocity, filling flow velocity, ejection fraction) than on the LAAS. Few studies have explored the relationship between LAAS and AF recurrence. The novelty of our research lies in investigating the relationship between LAAS and AF recurrence after RFCA, establishing a cutoff value for LAAS. Our study shows a higher risk of AF recurrence in patients with LAAS ≤ 5.67% or LAASR ≤ 2.70 s^−1.^ This may be explained by the fact that the LAA is more compliant than the LA and undergoes compensatory enlargement under LA pressure/volume overload, leading to earlier and more extensive myocardial damage [20]. Meanwhile, the extent of myocardial damage in the LA was more accurately assessed by LAAS measured via STE than by other parameters evaluated in this study. LAAS demonstrates higher accuracy, excellent reproducibility, and reduced susceptibility to confounders like pressure overload. Furthermore, Yang et al. found that LA and LAA GLS were independently associated with AF recurrence after RFCA [22]. Nevertheless, in terms of the optimal cut‐off value of LAA GLS (< 10.2%), it is inconsistent with our research results. The difference between the two cut‐off values may be due to the differences in patient composition and post‐processing software. Previous studies [29] have shown that the strain values measured by different ultrasound post‐processing software cannot be directly compared. In clinical applications, it is recommended that each center establish local reference values based on the same software. Thus, the clinical utility of LAAS as a predictive biomarker for AF recurrence, and especially its incremental prognostic value beyond conventional LAA parameters remains unclear and urgently needs clarification. In addition, a diagnostic model integrating clinical and echocardiographic data further confirmed the incremental predictive value of LAAS and LAASR for AF recurrence. Thus, incorporating 2D‐STE parameters into routine data and combining TTE and preoperative TEE can enable early detection of LA/LAA dysfunction in AF patients, aiding optimal selection for RFCA. Additionally, combining clinical data with LA routine parameters and 2D‐STE parameters can better predict the likelihood of postoperative AF recurrence when TEE is not suitable for the patient.

However, there are several limitations of this study. First, the sample size is relatively small and limited to a single medical center, which might affect the generalizability of the results. However, under the current study conditions, we included all eligible cases from our hospital and excluded those lost to follow‐up, resulting in a final cohort of 100 patients. To validate the findings, particularly the LAAS and LAASR cutoff values, larger sample sizes and multicenter research are warranted to validate the independent prognostic value of LAA strain in this setting before it can be broadly generalized. Second, due to the lack of specialized software available for assessing LAA strain, we used atrial strain software, which may have influenced the results. Third, the rate of AF recurrence may have been underestimated as asymptomatic AF events might not have been detected during the follow‐up period, potentially affecting the LAA strain–recurrence relationship and the optimal cut‐off determination. Regular and standardized assessment procedures help to minimize the magnitude of this bias. Fourth, 3D echocardiography was not employed to evaluate the volume and function of the LA and LAA, primarily due to the relatively lower image quality of 3D echocardiography compared to 2D imaging, as well as the time required for offline data analysis. In future studies, a larger patient cohort will be enrolled, and individuals with adequate image quality will be selected for 3D analysis. Fifth, this study assessed LA and LAA remodeling using ultrasound only, without employing LGE‐MRI to evaluate their structural remodeling. Since LA and LAA remodeling is not a specific manifestation of fibrosis, a purely functional assessment may fail to distinguish its underlying cause. Moreover, ultrasound parameters cannot localize or quantify the extent or distribution of fibrosis. Therefore, future studies should integrate both structural and functional parameters to validate the findings of this study.

## 5 Conclusion

LAAS and LAASR are potential predictors of AF recurrence, yet these results should be interpreted with caution, as they are derived from a single‐centre experience and are contingent upon the specific software used in this study. By incorporating 2D‐STE parameters into preoperative clinical data, along with TTE and TEE, we may be able to identify early on those patients with AF who have compromised LA/LAA function, thus select those who will benefit most from RFCA.

## Author Contributions

ZBY was a major contributor in writing the manuscript. QSH, LHR, and YDD participated in the data collection of the article. LYY, AX, and LGY participated in some revisions of the article. JSQ participated in the main image acquisition and content revision of the article. All authors read and approved the final manuscript. All authors contributed to writing, reviewing, and editing. All authors contributed to the article and approved the submitted version.

## Funding

The authors have nothing to report.

## Conflicts of Interest

The authors declare no conflicts of interest.

## Data Availability

The data that support the findings of this study are available on request from the corresponding author. The data are not publicly available due to privacy or ethical restrictions. Most of the data supporting this study are included in the article, the remaining data ta are not publicly available due to ethical restrictions.
